# The Results of Ultra-Processed Food Consumption on Weight Change: A Randomized Controlled Community Trial in a Health Promotion Program

**DOI:** 10.3390/nu17040638

**Published:** 2025-02-11

**Authors:** Mariana Souza Lopes, Patrícia Pinheiro de Freitas, Aline Cristine Souza Lopes

**Affiliations:** 1 Department of Nutrition, Universidade Federal da Paraíba, Campos I, s/n, Castelo Branco, João Pessoa 58050-585, Paraíba, Brazil; marianalopes.ufmg@gmail.com; 2 Department of Nutrition, Universidade Federal dos Vales do Jequitinhonha e Mucuri, Campus JK–Rodovia BR 367–Km 583, Diamantina 39100-000, Minas Gerais, Brazil; patpfreitas@gmail.com; 3 Department of Nutrition, Universidade Federal de Minas Gerais, Av. Alfredo Balena, 190, Santa Efigênia, Belo Horizonte 30130-100, Minas Gerais, Brazil

**Keywords:** overweight, obesity, Nova classification system, health services, Primary Health Care

## Abstract

Objective: Our objective was to evaluate the association between ultra-processed food (UPF) consumption and body weight change after participating in nutritional intervention. Design: Our study was a 12-month follow-up of participants in a randomized controlled community trial. Setting: Brazilian Primary Health Care. Participants: The participants were health promotion services users. Users in the control group (CG) performed the service’s usual intervention, while those in the intervention group (IG) additionally participated for seven months in nutritional intervention. Socioeconomic data, self-health, perception of time spent in health promotion services, and weight loss attempts were investigated. Food consumption was obtained by 24 h food recall and categorizing these in quartiles according to the Nova system of food classification. Weight was measured and changes in the 12-month period were calculated by subtracting the weight at follow-up from the baseline measurement. Results: Of the participants, 88.1% were females aged 56.7 ± 11.8 with 19.7 ± 15.3 months of participation in the service. In the fourth quartile (highest UPF consumption), the % contribution of calories per consumption of UPFs was 47.7%, with no differences between the IG and CG (*p* = 0.406). Adjusted after 12 months, when comparing those with lower consumption of UPFs (first quartile), individuals from the second, third, and fourth quartiles had positive weight variation. Respectively, these variations were as follows: 0.363 kg (95% CI: 0.038; 0.689; *p* = 0.029); 0.467 kg (95% CI: 0.159; 0.776; *p* = 0.003); and 0.389 kg (95% CI: 0.061; 0.717; *p* = 0.020, with no differences between IG and CG). Conclusions: The percentage contribution of calories from UPFs was associated with positive weight change, which contributes to the growing evidence of the relationship between UPFs and obesity.

## 1. Introduction

Overweight is a major risk for cardiovascular and other chronic diseases, and has quickly become a global epidemic with more than 1 billion obese people worldwide [1,2]. It has been consistently reported that ultra-processed food (UPF)-based diets cause excess calorie intake and weight gain and may facilitate the development of obesity [3,4].

UPFs are defined by the Nova system of food classification as products formulated mostly or entirely from food constituents not found in home cooking, which consist of culinary ingredients, such as fat, sugar, and salt [4,5]. In Brazil, the rise in UPF consumption has played a major role in the increase in the obesity epidemic [6]. In other countries, studies have also revealed the association between UPF consumption and body mass index (BMI) changes [7,8,9]. For example, a study with 110,260 participants, the NutriNet-Santé cohort study, observed BMI changes in all UPF consumption quartiles followed between 2009 and 2019; the weight gain appeared to be higher for participants in the second, third, and fourth quartiles compared to individuals from the first [8].

The magnitude of the obesity problem and its association with UPF consumption has led to the understanding that UPFs are important in preventing and reducing weight gain [2,4,10]. However, few studies of users of public health systems, thus far, have explored the role of UPF consumption in weight variation [11].

In Brazil, public health services are a priority locus for actions promoting health, food, and nutrition. Among them, the Health Academy Program (Programa Academia da Saúde—PAS, in Portuguese), one of the largest physical activity programs in the world, deserves special mention. The PAS units are spaces with infrastructure, equipment, and human resources for health promotion actions; they are located primarily in vulnerable areas [12,13]. PAS, as part of Brazilian Primary Health Care [12], offers a variety of health actions, at no cost, including physical exercises guided by a physical education instructor, for 60 min, three times a week [14]. However, nutritional activities are still not universal in the service. In Belo Horizonte, where this study was conducted, the lower prevalence of activities is associated with professional difficulties in expansion, primarily due to the high curative demands of health units other than the PAS.

Failure to explore the role of UPFs in the weight change in users of PAS could be the fault of repercussions of the evaluation of that policy—one of the largest public services promoting physical activity in the world [15]. In addition, since 2014, with the update of the Food Guide for the Brazilian Population, and more recently, guidelines for the treatment of obesity and other chronic diseases, all nutritional recommendations and proposals for nutritional interventions in Brazil are linked to the Nova classification. Therefore, investigating body weight changes from a Nova perspective can ensure the greater effectiveness of planned actions in the country and point out new paths to other countries [10,16].

Thus, the purpose of this study was to evaluate the association between ultra-processed food (UPF) consumption and body weight change after participating in nutritional intervention to promote fruit and vegetable consumption among the health promotion services of Brazilian Primary Health Care (PHC) users by participation in nutritional interventions.

## 2. Materials and Methods

### 2.1. Study Design and Samples

This study was based on data from the “Assessment of the impact of contextual and individual aspects, especially the consumption of ultra-processed foods, on the evolution of obesity in primary care users”, which was a randomized controlled community trial (RCCT) conducted with a representative sample of Brazilian health care services, between 2013 and 2017. The trial is registered with Brazilian clinical trials under RBR-8t7ssv URL: www.ensaiosclinicos.gov.br/rg/RBR-9h7ckx/ (accessed on 6 October 2024). Other details of this survey have been previously described [17].

The sample was drawn from a selection of PAS in Belo Horizonte, Minas Gerais, Brazil. Belo Horizonte, the capital of Minas Gerais, is the one of largest cities in South American. PAS is a national program of Brazilian Primary Health Care that offers a variety of health actions, at no cost, with the goal of expanding health care promotion and action [12,13].

The PAS units were randomly sampled from the eligible facilities, which included all 42 units operating at the time of study (2012). These units had to meet the following criteria: they opened in the morning, were located in an area of medium and high vulnerability to health issues (predominant period and focus of operation of PAS, respectively), and had not been the subject of research related to food and nutrition in the previous 24 months [17]. This sample was stratified by the nine administrative districts of the municipality. Two PAS units in each stratum, paired according to health vulnerability, were selected, based on the criteria used for the implementation of the health service in the municipality [17].

Eighteen PAS units were selected and divided between the control group (CG) (*n* = 9) and the intervention group (IG) (*n* = 9). The selected units represented medium- and high-vulnerability areas in the municipality, with a reliability of 95% and an error rate of less than 1.4% [17].

For this study, we included all PAS users aged 20 years or older who habituated the activities within the PAS (participation in physical exercise in the preceding month). We excluded users who were pregnant and with cognitive impairment [17].

### 2.2. Data Collection

Data were obtained by trained dietitians and students. Data at baseline (M0) and 12 months later (M1) were obtained from face-to-face interviews.

The questionnaires were based on national surveys and on the previous experience of the research group. Data collection was preceded by a pilot study. The questionnaire included sociodemographic information (sex, age, income, education, occupation, and marital status), self-health perceptions, physical activity, attempts to lose weight, time of participation at PAS, dietary assessment, and weight (in kilograms, kg) using a digital scale. After 12 months, we re-evaluated weight [17]. This was in accordance with the national recommendations for the collection and analysis of anthropometric data [18].

### 2.3. Dietary Assessment

At baseline, food consumption was investigated using the 24 h food recall instrument (R24h) from the Multiple-Pass method [19]. Participants were asked about all food and drink consumed both at home and outside on the previous day and their respective household measurements and preparation methods. Participants answered two R24h on alternate days. The days were randomly selected to guarantee a balanced representation of all days of the week. The foods mentioned in R24h were listed with the help of the Brasil-Nutri Software version 1.2 developed by the Brazilian Institute of Geography and Statistics for the National Food Survey.

Then, the self-reported measurements of food were transformed into grams or milliliters using food consumption assessment tables and manuals and industrialized food labels and measurements (standardizing/weighing [19,20]. Consequently, consumption in grams or milliliters was converted to kilocalories from the national food composition table [21].

Next, all food items were classified according to the Nova food classification system [5]. Nova classifies foods into four groups: (1) unprocessed or minimally processed foods (e.g., fruits or vegetables, grains, pasteurized milk, and meat); (2) processed culinary ingredients (e.g., sugar, oils, and salt); (3) processed foods (e.g., cheese, fruits in syrup, and canned foods); and (4) ultra-processed foods (e.g., soft drinks, packaged snacks, pre-prepared frozen or shelf-stable dishes). However, UPFs were the only focus of this study. More information regarding the Nova classification can be found elsewhere [5,16].

Imputation for the missing data on food consumption in the RCCT rounds was carried out: baseline (*n* = 5) and follow-up (*n* = 1178). The first step was to identify outliers of food consumption. Values of total energy consumption considered very low (<500 kcal) or very high (>7000 kcal) (*n* = 65) were identified as outliers and transformed into missing values. The second step was to assess the imputation of food consumption by the value of the observed round (Round 1 = baseline and Round 2 = reassessment after 12 months). Thus, we imputed the missing value from the information of the round that had information.

Therefore, those who did not have information in Round 1 were imputed by Round 2, and those who did not have information in Round 2 were imputed by Round 1. In this step, ten possible values were generated, and we selected the average of these as the value imputed. When the individual had no value for either Round 1 or Round 2, we used the overall mean of observations from that period. When the imputation value was negative, the minimum positive value estimated after imputation was assigned. The third step was to perform the imputation by the value of the observed round plus the sociodemographic variable (gender, age, and education). To this end, we imputed the missing value from the information of the round that had information plus sociodemographic variables. In this process, ten possible values were generated from the imputation, and we selected the average of those as the imputed value.

We emphasize that when the individual had no value for either Round 1 or Round 2 or had no information for the education variable, we used the general average of the observations in that period. And when the value of the imputation was negative, we assigned the minimum positive value estimated after imputation. Finally, step 4 was carried out and characterized by descriptive data analysis. For this purpose, Kernel density plots were created to compare original and imputed data.

For analyses of UPF consumption, we used percent in quartiles of the contribution of energy from these UPFs concerning the total energy value of the diet (%), using the following formula: (kcal UPFs × 100)/kcal total. The first quartile corresponded to the lowest consumption and the last to the highest consumption for each food group.

### 2.4. Outcome Assessment

The outcome of this study showed the body weight variations after 12 months of follow-up. The weight variations were treated as continuous variables obtained for a difference in body weight (kg) after 12 follow-ups (∆kg). Weight variations were assessed by subtracting the weight at 12 months of follow-up from baseline information: [weight variations (∆kg) = (weight at 12 months − weight at baseline)]. Thus, a negative variation demonstrated weight loss and a positive variation demonstrated weight gain.

An intention-to-treat analysis was employed for body weight using information from individuals (sex, age, education, and other weight measures) to estimate missing data. For this purpose, the same imputation method of consumption data was used. Subsequently, the estimated weight value for missing information was calculated as the average of ten random values estimated by the imputation package in Stata. The imputed values for weight were compared by the mean and standard deviation and the probability density distribution. The final result was satisfactory, without major differences between the follow-up reporting and the probability distribution before and after imputation.

### 2.5. Covariates

The covariates analyzed were age (years), sex (female/male), education (years), time of participation in the PAS (months), occupation (retired, unemployed, pensioner, and others), marital status (divorced, single or widowed), physical activity (weekly frequency and hours the activity was performed per day), following a diet to lose weight (yes/no), and participation in nutritional intervention (yes/no). The time of participation in the PAS was calculated from the date of the first interview in the research and the date of entry of the interviewee in the program provided by the City Hall.

### 2.6. Nutrition Intervention

This was a nutritional intervention to promote adequate and healthy eating, especially the consumption of fruits and vegetables. The CG participants participated in routine service activities, which included light physical activity three times a week for 60 min. The IG additionally had seven months—this period being between the M0 and the M1—of collective intervention to encourage the consumption of fruits and vegetables with a view to reducing UPF consumption and replacing these foods with FV whenever possible [22,23]. To obtain this answer, participants were asked about the weekly frequency and time spent practicing physical activity.

The collective nutritional intervention was planned by an interdisciplinary team comprising nutritionists and educators. It aimed to increase individuals’ confidence in their ability to increase FV consumption when faced with obstacles (self-efficacy) and to increase awareness of the benefits of a healthy diet while minimizing the factors against change (decisional balance) [22,23].

The theoretical foundation of the intervention was built based on the components of problematizing dialogic pedagogy by Paulo Freire and the transtheoretical model [23,24]. These helped in the planning and implementation of actions, because they enabled the classification of individuals according to perception, readiness, attitude, and motivation for change. For such an assessment, the transtheoretical model includes four pillars: stages of change, processes of change, self-efficacy, and decisional balance. Change stages define the change action planning phases. Change processes indicate how change occurs during different stages. Self-efficacy reveals the individual’s confidence in their ability to achieve the desired behavior in the face of obstacles and decision balance involves increasing awareness of the benefits of healthy eating while minimizing factors that are contrary to change [22].

The participants were grouped by stages for FV consumption in the following subgroups: pre-action (including pre-contemplation and contemplation stages: individuals are not ready to change, but require action to shape their motivation); preparation (individuals are ready to change their behavior within 30 days); and action (including action and maintenance stages: individuals are capable of immediate changes, but the prevention of relapse and consolidation of gains are required) [23,25].

The educational strategies used included workshops, motivational messages via postcards, telephone calls to invite and remind participants of the group sessions, environment-based activities (e.g., movies and cooking competitions), education panels, and the delivery of informative material [23]. The intervention varied across different stages of change with respect to FV intake and consisted of four educational group sessions with up to 20 participants, three postcards, and three environment-based activities for each group, plus one handout with informative material. The topics addressed were related to food portions, nutritional information, obstacles to changing FV consumption, difficulties in accessing quality FV, and others [23]. Details on nutritional interventions have been detailed elsewhere [22,23].

This type of intervention did not allow blinding participants and investigators for the study. However, the intervention was performed by all participants in the units of the IG. In all analyses, participants were kept in their original groups.

### 2.7. Statistical Analysis

First, we estimated the distribution of total dietary energy intake according to UPF consumption. Then, the percentage of UPF contribution from the diet was divided into consumption quartiles. Then, we examined how quartiles of UPF consumption varied according to the characteristics of the study population. For this, we used an analysis of the linear trend test or χ^2^ tests when appropriate.

We analyzed differences in weight change according to baseline, and a *t*-test was applied. In addition, to examine the extent to which individual- and area-level factors explain weight variations, we estimated a linear regression model controlling for the PAS cluster, having as outcome the measures of weight variations. Covariates were added based on the conceptual model. The models were adjusted for time of participation at PAS (months).

Multiple regression analysis was used to evaluate associations between the consumption of UPFs and weight change (kg). Models were adjusted for group (intervention) and weight at baseline (Model 1), in addition to age (years, continuous variables), sex, education (years, continuous variables), and occupation (Model 2). Based on Model 2, we additionally adjusted for attempts to lose weight and time in PAS (continuous months) (Model 3).

Statistical significance was attributed when *p* values were less than 0.05. Analyses were performed using Stata. Mean and standard deviations were calculated for all continuous variables, and frequency was used to describe qualitative variables

## 3. Results

**Figure 1:**
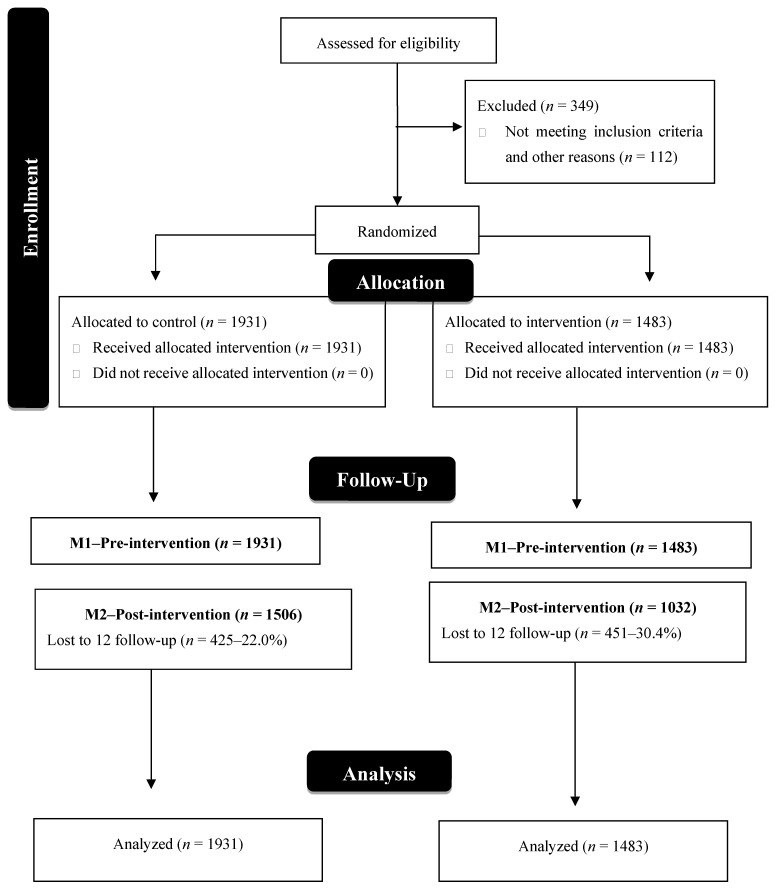
Flowchart of study participants of Fruit and Vegetable Randomized Controlled Community Trial. Belo Horizonte-MG, Brazil. 2013–2017.

Data at baseline and at 12 months of follow up are presented for all 3414 participants, who were allocated to the control (*n* = 1931) and intervention groups (*n* = 1483) (Figure 1).

We investigated 3414 individuals, 88.1% female, aged (mean ± SD) 56.7 ± 11.8, 7.2 ± 4.1 years of education, and with 19.7 ± 15.3 months of participation in PAS, to determine the differences between the IG and the CG. More than half (61%) of the participants had recently tried to lose weight (Table 1).

**Figure 2:**
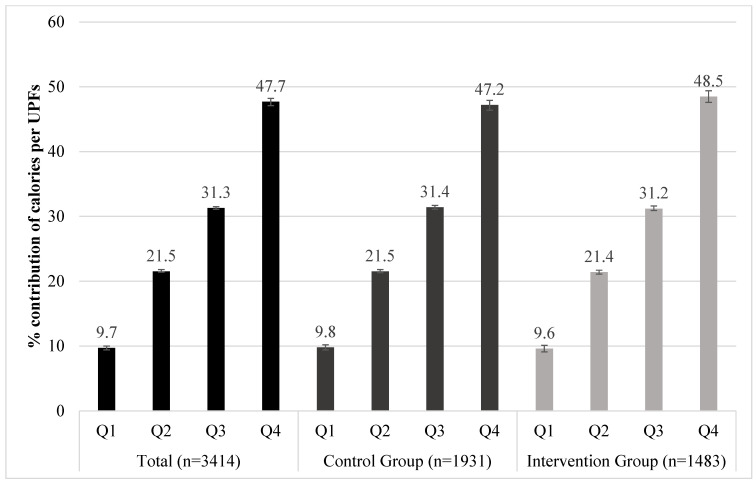
Baseline percent contribution of calories (Kcal) per ultra-processed food consumption in a Fruit and Vegetable Randomized Controlled Community Trial. Belo Horizonte-MG, Brazil. 2013–2017. Notes: UPFs = ultra-processed foods. First quintile corresponds to the lowest and quintile five to the highest intake of ultra-processed foods. % contribution of calories per UPF: Q1 = 9.7%, Q2 = 21.4, Q3 = 31.3%, and Q4 = 47.8%. Chi-square test, *p* value = 0.406.

The general control and intervention group baseline characteristics, by quartiles of percent contribution of calories per UPF, are presented in Figure 2 and Table 1.

The mean contribution of calories per UPF consumption at the last quartile at baseline was 47.7% (CI 95%: 47.2% to 48.3%) vs. 9.7% (CI 95%: 9.4% to 10%) at first quartile, without any difference between the participants in the control and intervention groups (*p* = 0.406) (Figure 2).

With regard to differences between the total sample GC and GI, when we stratified UPFs by quartiles of consumption, we identified that individuals in the CG with higher consumption of UPFs (last quartile) were older (55.3 ± 12.1 y vs. 52.9 ± 12.4 y; *p* = 0.021), had a higher level of education (8.1 ± 4.1 y vs. 7.8 ± 3.9 y; *p* = <0.001), had a higher income (BRL 1017.5 ± 874.0 vs. BRL 830.0 ± 668.9; *p* = 0.001), and a longer participation time at PAS (20.7 ± 16.2 months vs. 15.5 ± 13.5 months; *p* < 0.001) in comparison to their counterparts in IG. In addition, they had a lower prevalence of weight loss attempts (62.3% vs. 64.2%; *p* = 0.047) (Table 1).

In Table 2, the mean of weight change is presented according to the users’ quartile of contribution of calories per UPF consumption. Among individuals in the fourth quartile with the highest consumption of UPFs, there was a positive variation in weight, 0.178 kg (CI 95%: −0.081 to 0.436), with a difference between the CG and IG of (CG = −0.096 kg; CI 95%: −0.459; 0.267 vs. IG = 0.531 kg; 95% CI: 0.169; 0.894; *p* value = 0.018).

After adjusting, when comparing those with a lower consumption of UPFs (first quartile), individuals from the second, third, and fourth quartiles had positive weight variations, which were, respectively, as follows: 0.363 Kg (95% CI: 0.038 to 0.689; *p* = 0.029); 0.467 Kg (95% CI: 0.159 to 0.776; *p* = 0.003), and 0.389 Kg (95%CI: 0.061 to 0.717; *p* = 0.020), with no difference between the CG and IG (Table 3).

## 4. Discussion

Greater UPF consumption at baseline was associated with weight gain among users of the Brazil’s Unified Health System health promotion program after 12 months of follow-up. We did not identify differences in weight gain according to participation in the nutritional intervention.

The positive relation of UPF consumption at baseline and weight variation corroborates previous findings [8,26,27]. Previous research conducted in France with 110,260 adult participants (≥18 years old, mean baseline age = 43.1 [SD 14.6] years; 78.2% women) from the NutriNet-Santé cohort (2009–2019) showed a positive association between UPF consumption and gain in BMI, with an absolute increment of ten percentage points in the UPFs in the diet. In the same direction, a prospective cohort including 348 748 men and women aged 25–70 years from nine European countries in the European Prospective Investigation into Cancer and Nutrition (EPIC) study showed that higher UPF consumption (per 1 SD increment) was positively associated with weight gain (0.12 kg/5 years, 95% CI 0.09 to 0.15) [7]. A similar trend is observed in studies on other continents [26]. An open-label, randomized, crossover study conducted from August to October 2022 at the University of Tokyo Hospital, Tokyo, Japan, reveled gains of 1.1 kg more weight (95% confidence interval 0.2 to 2.0; *p* = 0.021), which was explained by UPF consumption [28].

In Brazil, a similar trend was observed in a cohort of civil servants of Brazilian public academic institutions. After adjustment, the fourth quartile (>30.8%) and first quartile (<17.8%) of UPF consumption were associated with 27% and 33% greater risk of weight gain and increased waist circumference, respectively [27]. Our results significantly extend these previous findings by including users’ Unified Health System.

The need for knowledge about the public health services offered to Unified Health System users in Brazil is relevant for health professionals, managers, and the population due to its potential for improving the quality of services offered and its success in improving the health of the population. Furthermore, such knowledge can guide the formulation and implementation of health policies with the optimization of efforts not only in Brazil, but also in other countries with universal health systems.

It has been previously established that UPFs show a greater energy density of saturated fat, trans fats, and sugar, and less fiber, protein, and potassium, when compared with non-processed foods (e.g., fruit and vegetables) [4,29]. In addition, they are often consumed in larger portions [29,30], highly palatable, and convenient [4,31]. Furthermore, they often have an appealing advertising presence [32,33,34] and are probably addictive, directly driving forward excessive patterns of food intake and contributing to obesity [35]. Other mechanisms are explained via UPF additives (e.g., emulsifiers), which can lead to the disruption of the intestinal mucosal barrier and produce chronic inflammation that is a risk factor for obesity [36]. However, it is also possible that UPF intake lessens unprocessed or minimally processed food, for example fruit and vegetables (obesity protectors), from the daily diet. Other studies must be carried out to clarify this hypothesis and verify whether the variation in UPF consumption, between baseline and reassessment, was associated with weight change.

The previous statement can be helpful in realizing the second result: the intervention to promote the consumption of unprocessed food, such as FV, seems to have not been important in preventing weight gain among its participants with a high consumption of UPFs. We hypothesize that working with only one of the Nova groups may not be sufficient to achieve positive results in weight loss. The results of nutritional intervention in FV consumption are well known, as evidenced in another analysis, which showed that the increase in FV intake was 23.4 g/d (95% CI: 6.7, 40.0; *p* = 0.01) and fruit intake, 17.3 g/d (95% CI: 5.1, 29.4; *p* = 0.01); this was greater in the IG among participants in the lowest baseline intake [22]. On the other hand, studying other outcomes, such as the nutritional profile of the diet and weight loss, a loss of intervention effect was also observed [11,29]. However, these studies are conclusive in indicating the importance of nutritional interventions, especially in health services, for the promotion and maintenance of health.

This leads us to believe that positive changes in FV consumption are not necessarily associated with a reduction in UPF consumption, and may not be sufficient for weight loss. One interesting path may be to deepen the peculiarity of this Nova group. Unlike fruits and vegetables, UPFs are associated with indiscriminate marketing and can cause addiction. This may predispose individuals to more frequent and excessive consumption. Future studies should be conducted to clarify the causal pathways that explain links between food processing and adiposity. This evidence is relevant to inform policy makers.

The limitations and strengths of the present study should be considered when interpreting findings. One limitation was the absence of data about weight variations throughout life, including variables such as the heaviest weight they ever had and data about bariatric surgery or use of specific weight loss medications. In addition, we have investigated consumption only at baseline, not exploring its variations.

Caution is required when extrapolating our results to the general population, as participants attend a health promotion service, which might make them more aware of their health, including nutritious eating. However, we believe that the results of this study can be extrapolated to other populations of the Primary Health Care users of the Brazilian Unified Health System. It is estimated that, in Brazil, more than 80% of the population uses the Unified Health System, which indicates its extension in the country and can minimize some of the limitations presented. In addition, internal validity was further strengthened by the prospective study design and direct measures of anthropometrics. UPF consumption was assessed according to the Nova classification system by using data from multiple 24 h dietary recalls associated with a home measurement kit, the least biased self-report assessment available; adjustments for several confounding factors were made.

The present study has several strengths, and given the longitudinal study design, causal inferences were analyzed. To the best of our knowledge, this is one of the first national studies with users of the Brazil’s Unified Health System to longitudinally reveal the association between the consumption of UPFs and variations in weight, regardless of nutritional intervention. The analyses were based on the Nova food classification system, which has been recognized by international agencies as a relevant approach for linking dietary intake and weight variation and obesity. This strengthens the valuable information that UPF consumption is associated with weight variation and supports the potential role of UPFs in contributing to obesity in Brazil’s Unified Health System users.

## 5. Conclusions

In conclusion, greater UPFs consumption at baseline was associated with weight gain in users of Brazilian PHC. The nutritional intervention, based on encouraging the consumption of unprocessed foods, does not seem to be sufficient in preventing weight gain due to the excessive consumption of UPFs. This may indicate that the peculiarities involving the consumption of UPFs, such as unfair marketing, prices, and compositions, need to be worked on with an outlook toward shifting the consumption of UPFs to fruits and vegetables.

## Figures and Tables

**Table 1:** Characteristics of the study population according to quartiles (Q) of baseline percent contribution of calories (Kcal) per ultra-processed food (UPF) in a Fruit and Vegetable Randomized Controlled Community Trial. Belo Horizonte-MG, Brazil. 2013–2017.

Variables	Total	Control Group (*n* = 1931)				Intervention Group (*n* = 1483)				*p* Value Between Group
		Q1	Q2	Q3	Q4	Q1	Q2	Q3	Q4	
Age (yrs), mean ± SD	56.7 ± 11.8	58.9 ± 10.7	57.9 ± 10.6	56.3 ± 11.7	55.3 ± 12.1	59.4 ± 11.6	57.5 ± 11.8	54.9 ± 12.1	52.9 ± 12.4	0.021 *
Education (yrs), mean ± SD	7.2 ± 4.1	6.9 ± 4.2	7.4 ± 4.0	7.4 ± 4.0	8.1 ± 4.1	5.6 ± 0.9	7.1 ± 4.2	7.2 ± 4.1	7.8 ± 3.9	<0.001 *
Income per capita ^&^, USD mean ± SD	374.8 ± 343.4	365.2 ± 355.0	398.4 ± 340.2	375.4 ± 328.0	431.1 ± 353.0	354.8 ± 474.5	359.3 ± 263.2	340.8 ± 262,5	351.7 ± 283.4	0.001 *
Sex, %										
Female	88.1	84.0	88.7	91.8	90.8	81.3	86.5	92.0	88.4	<0.001 **
Men	11.9	15.9	11.3	8.2	9.2	18.7	13.5	7.9	11.6	
Marital status, %										
Married	61.6	63.1	61.5	64.2	49.0	64.9	56.7	58.9	63.4	0.086 **
Divorced	8.3	8.2	7.3	6.1	9.8	6.7	8.43	10.4	10.2	
Single	14.1	12.3	12.1	14.7	17.3	13.6	14.6	14.2	14.5	
Widowed	16.0	16.4	19.1	14.9	13.9	14.9	20.2	16.4	11.8	
Occupation, %										
Housewife	28.7	27.6	31.8	30.7	24.3	26.9	26.4	32.0	29.6	<0.001 **
Retired or pensioner	36.7	47.7	36.2	34.2	34.3	44.9	41.3	33.7	26.3	
Unemployed	2.0	1.9	1.6	0.4	2.1	2.8	2.0	1.6	4.0	
Employed	32.6	27.8	30.4	34.6	39.3	25.4	30.3	32.6	40.0	
Self-health perceptions, %										
Very poor/poor/regular	28.3	34.5	27.2	26.6	24.7	31.3	28.4	29.6	24.5	0.193 **
Good/very good	71.7	65.5	72.8	73.4	75.3	68.7	71.6	70.4	75.5	
Recent weight loss attempt, %	61.0	57.8	56.7	63.3	62.3	56.7	62.1	66.0	64.2	0.047 **
Time of participation in PAS (months), mean ±SD	19.7 ± 15.3	21.6 ± 16.1	21.6 ± 16.1	21.7 ± 16.3	20.7 ± 16.2	18.4 ± 13.8	18.1 ± 14.3	17.8 ± 13.8	15.5 ± 13.5	<0.001 *

Notes: data are expressed as arithmetic mean ± standard deviation (SD) if not stated otherwise. First quintile corresponds to the lowest and quintile five to the highest intake of ultra-processed foods. A total of 2 missing data items for education; 4278 missing data items for income per capita. % contribution of calories per UPF: Q1 = 9.7%, Q2 = 21.4, Q3 = 31.3%, and Q4 = 47.8%. * Linear trend test; ** χ^2^ tests when appropriate; ^&^ in 2013, USD 1 was BRL 2.36.

**Table 2:** Mean of weight change (kilograms) after 12 months of follow-up according to users’ percent contribution of calories (Kcal) per ultra-processed food consumption in a Fruit and Vegetable Randomized Controlled Community Trial. Belo Horizonte-MG, Brazil. 2013–2017.

% Contribution of Calories per UPF	Total (*n* = 3414)		Control (*n* = 1931)		Intervention (*n* = 1483)		*p* Value
	Mean of Weight Change (Δkg)	95% CI	Mean of Weight Change (Δkg)	95% CI	Mean of Weight Change (Δkg)	95% CI	
Q1 (lowest)	−0.239	−0.452; −0.026	−0.220	−0.455; 0.014	−0.262	−0.638; 0.114	0.848
Q2	0.107	−0.161; 0.376	0.111	−0.281; 0.505	0.100	−0.238; 0.440	0.968
Q3	0.242	0.016; 0.469	0.261	−0.040; 0.563	0.218	−0.126; 0.562	0.851
Q4	0.178	−0.081; 0.436	−0.096	−0.459; 0.267	0.531	0.169; 0.894	0.018

**Table 3:** Difference in body weight gain (kg) over 12 months according to baseline users’ percent contribution of calories (Kcal) per ultra-processed food consumption in a Fruit and Vegetable Randomized Controlled Community Trial. Belo Horizonte-MG, Brazil. 2013–2017.

Variables	MODEL 1			MODEL 2			MODEL 3		
	Coef.	*p*-Value	CI (95%)	Coef.	*p*-Value	CI (95%)	Coef.	*p*-Value	CI (95%)
% contribution of calories per UPF									
Q2	0.371	0.025	0.471; 0.697	0.363	0.029	0.038; 0.689	0.363	0.028	0.038; 0.688
Q3	0.480	0.002	0.174; 0.786	0.467	0.003	0.159; 0.776	0.459	0.004	0.151; 0.768
Q4	0.440	0.009	1.091; 0.771	0.389	0.020	0.061; 0.717	0.381	0.024	0.051; 0.711
Group	0.153	0.212	−0.087; 0.392	0.156	0.202	−0.083; 0.395	0.150	0.207	−0.083; 0.384
Weight at baseline	−0.055	<0.001	−0.082; –0.276	−0.058	<0.001	−0.087; −0.030	−0.061	<0.001	−0.095; −0.027
Age (years)				−0.001	0.863	−0.015; 0.0130	−0.001	0.998	−0.015; 0.015
Sex				0.433	0.036	0.029; 0.839	0.461	0.041	0.018; 0.905
Education (years)				0.018	0.195	−0.009; 0.046	0.019	0.185	−0.009; 0.047
Retired or pensioner				−0.083	0.693	−0.428; 0.263	−0.081	0.647	−0.428; 0.266
Unemployed				0.347	0.402	−0.465; 1.159	0.328	0.426	−0.481; 1.138
Employed				0.252	0.159	−0.099; 0.604	0.260	0.153	−0.097; 0.617
Attempt to lose weight							0.184	0.409	−0.248; 0.609
Time in PAS (months)							−0.001	0.770	−0.009; 0.007

## Data Availability

The original contributions presented in the study are included in the article, further inquiries can be directed to the corresponding author.
